# How to apply evidence-based practice to the use of artificial intelligence in radiology (EBRAI) using the data algorithm training output (DATO) method

**DOI:** 10.1259/bjr.20220215

**Published:** 2023-03-22

**Authors:** Brendan S Kelly, Conor Judge, Siobhan Hoare, Gabrielle Colleran, Aonghus Lawlor, Ronan P Killeen

**Affiliations:** 1 St Vincent’s University Hospital, Elm Park, Dublin, Ireland; 2 Insight Centre for Data Analytics, University College Dublin, Belfield, Dublin, Ireland; 3 Wellcome-HRB Irish Clinical Academic Training (ICAT), Dublin, Ireland; 4 HRB-Clinical Research Facility, NUI Galway, Galway, Ireland; 5 CHI @ Temple Street, Dublin, Ireland

## Abstract

**Objective:**

As the number of radiology artificial intelligence (AI) papers increases, there are new challenges for reviewing the AI literature as well as differences to be aware of, for those familiar with the clinical radiology literature. We aim to introduce a tool to aid in this process.

**Methods:**

In evidence-based practise (EBP), you must Ask, Search, Appraise, Apply and Evaluate to come to an evidence-based decision. The bottom-up evidence-based radiology (EBR) method allows for a systematic way of choosing the correct radiological investigation or treatment. Just as the population intervention comparison outcome (PICO) method is an established means of asking an answerable question; herein, we introduce the data algorithm training output (DATO) method to complement PICO by considering Data, Algorithm, Training and Output in the use of AI to answer the question.

**Results:**

We illustrate the DATO method with a worked example concerning bone age assessment from skeletal radiographs. After a systematic search, 17 bone age estimation papers (5 of which externally validated their results) were appraised. The paper with the best DATO metrics found that an ensemble model combining uncorrelated, high performing simple models should achieve error rates comparable to human performance.

**Conclusion:**

Considering DATO in the application of EBR to AI is a simple systematic approach to this potentially daunting subject.

**Advances in knowledge:**

The growth of AI in radiology means that radiologists and related professionals now need to be able to review not only clinical radiological literature but also research using AI methods.

Considering Data, Algorithm, Training and Output in the application of EBR to AI is a simple systematic approach to this potentially daunting subject.

## Introduction

The workload associated with medical imaging has greatly increased both in terms of volume and complexity. However, the clinicians trained in expert interpretation of this avalanche of data has failed to keep pace with demand.^1^ Artificial intelligence (AI) applications have been suggested as a possible solution to this supply–demand issue.^2^ While there has been a recent explosion in the radiology AI literature,^3^ there is a paucity of high-level evidence for the implementation of AI into clinical radiology. This has been termed the “AI Chasm”.^4^ The evidence-based practise (EBP) paradigm is well-established^5^ and uses the Ask, Search, Appraise, Apply, and Evaluate method. Both bottom-up and top-down approaches are described for the application of EBP to radiology (EBR).^6^ The top-down method is based on guidelines being “pushed” down from academic centres, while the second method attempts to meld international best practise with local expertise and available resources. The decision to undertake a research project in AI or being involved in purchasing a product that uses AI are examples of opportunities to use bottom-up EBR techniques.

Patient intervention comparison outcome (PICO) is a well-established tool in the EBP paradigm. For evidence-based radiology artificial intelligence (EBRAI), we additionally propose the considering DATO (data algorithm training output) to access the use of AI to answer a given research question.

### DATO stands for

Data: Here, we consider how the data were curated and issues of quality and quantity including: ethics, data access, querying data, deidentification, transfer, quality control, structure, ground truth, labelling.^7^

Algorithm: An algorithm is a process or set of rules followed to achieve a goal. The algorithm chosen therefore is a direct consequence of the task. Major common tasks in radiology AI include segmentation, identification, classification, regression and prediction. Different algorithms suit different tasks, and both must be considered in this section.

Training: This incorporates model development and model evaluation. The model may be trained and tested on internal data or might include some level of external validation. Using external validation has a higher level of scientific rigour as it can expose overfitting and increase the potential for generalisability.

Output: Ultimately, there will be some kind of output from the model. This might be a diagnosis, prognosis, or clinical measurement etc. The output must be applicable to the users’ local needs to be useful clinically.

**Figure 1.**
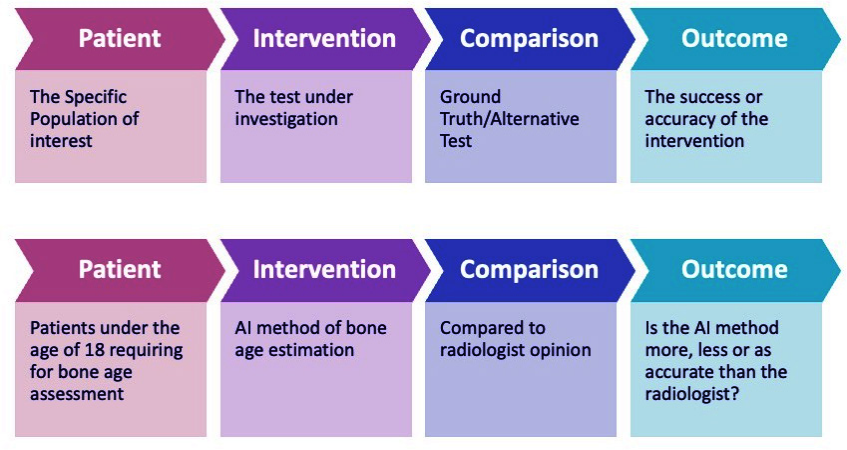
The use of PICO to ask an answerable question. AI, artificial intelligence; PICO, patient intervention comparison outcome.

**Figure 2.**
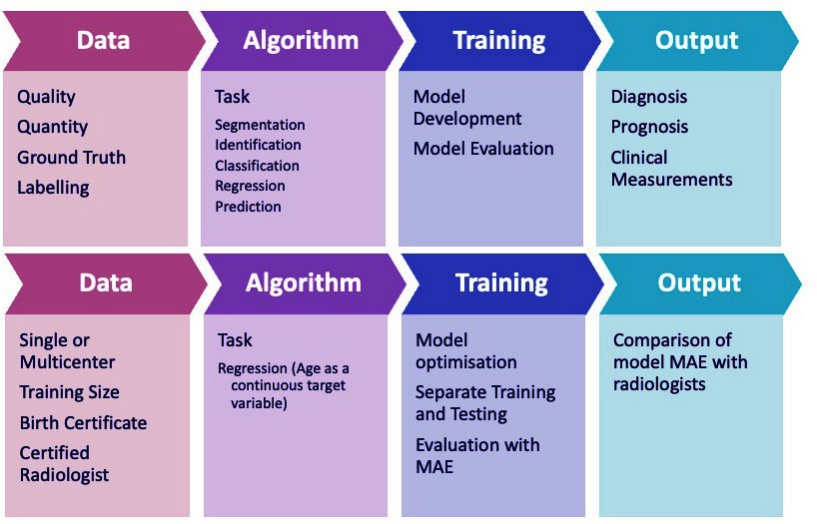
The use of DATO in the application of AI to answer the question. AI, artificial intelligence; DATO, data algorithm training output.

A comparison of PICO and DATO showing how this information can be incorporated into each subheading is illustrated in Figures 1 and 2. An explanation of the terminology used above is available in Table 1.

**Table 1.** Glossary of terms

Glossary of terms	
Algorithm	A process or set of rules followed to achieve a goal.
Classification	A general process of categorisation assigning labels to samples
Data	A collection of quantitative and qualitative variables
Data access	The availability of medical data is restricted to authorised professionals such as physicians, technologists, PACS managers, and clinical scientists. Enabling AI developers to access these data can be a difficult task that involves several stages.
Data de-identification	Both HIPAA and the European General Data Protection Regulation mandate the proper de-identification of retrospectively and prospectively collected data. Sensitive information that needs to be de-identified includes but is not limited to name, medical record number, and date of birth.
Data ethics	Before medical data can be utilised for developing a research or commercial AI algorithm, approval from the local ethical committee is necessary. An institutional review board is responsible for assessing the potential risks and benefits of the study to the patients.
Data labelling	Similar to annotation this is the answer or result in supervised learning
Data query	The process of searching for medical images and clinical data
Data structure	Organising and storing data in homogenised and machine-readable formats
Data transfer	Data are often transferred to either a local data storage (in the case of a single-centre study) or an external data storage (in the case of a multicentre study or commercial AI development). Typically, data are stored on an on-premise server; however, with the emergence of cloud-based technology, data are increasingly being stored in the cloud.
External validation	To accurately assess generalisability, independent validation of results in a data set obtained from a different institution is preferable to internal validation.
Ground truth	The term “ground truth” typically refers to information obtained through direct observation, such as biopsy or laboratory results. In the case of medical imaging, image labels are annotations performed by medical experts, such as radiologists, and can be considered ground truth if imaging is the reference standard.
Identification	Detection and or localisation of one or more structures in an image
Model	A “model” refers to a mathematical or computational representation of a system, process, or phenomenon that can be used to make predictions or decisions
Model development	In machine learning, a model is typically trained on a data set to learn patterns and relationships in the data, which can then be used to make predictions on new, unseen data.
Model evaluation	The process of measuring the performance of a machine learning model.
Output	The result or prediction produced by a machine learning model for a given input.
Prediction	The process of using a machine learning model to estimate or infer an output value based on one or more input values.
Quality control	The process of evaluating and ensuring the accuracy, reliability, and consistency of the input data and results outputted by a machine learning model or AI system.
Regression	A type of machine learning task where the goal is to predict a numerical value, or a continuous output variable
Segmentation	The process of dividing an image, video, or other data into smaller, more meaningful parts or regions.

AI, artificial intelligence.

We will use the example of a local research team interested in undertaking a research project involving the automated assessment of bone age in children on hand/wrist radiographs for the application of the DATO method. However, the process would be similar in the scenario where a department were considering purchasing a product that used AI for the same purpose.

Radiographic bone age assessment is an important component of the diagnostic workup for a variety of paediatric endocrine, metabolic, and growth disorders.^8,9^ There are many different methodologies employed in bone age estimation, however the most commonly used is Radiographic Atlas of Skeletal Development of the Hand and Wrist by Greulich and Pyle (GP). The Tanner-Whitehouse method of bone age assessment is a more reliable alternative to GP; however, it is relatively labour intensive and time-consuming.^8,9^ As these methods are time- and labour intensive as well as monotonous and repetitive, they have become a popular early use case for AI.^10^ Automated commercial methods exist with good accuracy but have an associated cost and are trained on historical data, which can limit their generalisability.

## Methods

### Ask

PICO is an established tool for asking an answerable question. Our example question is:

 “What type of AI is best at assessing bone age?”

Figure 1 shows how we use the PICO tool to turn this into the answerable question: “In paediatric patients who require bone age assessment with hand/wrist radiographs how do AI models compare with human performance using birth certificate as ground truth?”

However, to answer this question using AI, we can also consider what data would be needed, what algorithm would be appropriate, what training is required and what model output would be useful (Figure 2).

### Search

The process of searching the literature usually involves searching primary literature with one or more of the established electronic databases.^11^ The AI literature has the additional complication that preprint repositories such as arXiv are widely used.  It is also important to include search terms relevant to the AI literature via the “AND” operator. A suggested set of search terms is provided in Table 2. For our example, we have built on an ongoing systematic review of the paediatric AI radiology literature. Details of our full search strategy is available in a published protocol.^3,12^

**Table 2.** List of search terms

Artificial intelligence	Radiology	Pediatrics
(Artificial intelligence[Title/Abstract])OR (Machine learning[Title/Abstract])OR (Support vector machine[Title/Abstract])OR (SVM[Title/Abstract])OR (CNN[Title/Abstract])OR (RNN[Title/Abstract])OR (LSTM[Title/Abstract])OR (ResNet[Title/Abstract])OR (DenseNet[Title/Abstract])OR (Unet[Title/Abstract])OR (U-net[Title/Abstract])OR (DNN[Title/Abstract])OR (Neural network*[Title/Abstract])OR (Convolutional network*[Title/Abstract])OR (Deep learn*[Title/Abstract])OR (Semantic segmentation[Title/Abstract])OR (Ensemble[Title/Abstract])OR (Classification tree[Title/Abstract])OR (regression tree[Title/Abstract])OR (probability tree[Title/Abstract])OR (nearest neighbo*[Title/Abstract])OR (fuzzy logi*[Title/Abstract])OR (random forest[Title/Abstract])OR (kernel[Title/Abstract])OR (k-means[Title/Abstract])OR (naive bayes[Title/Abstract])	(X-ray*[Title/Abstract])OR (X-ray*[Title/Abstract])OR (Radiography[Title/Abstract])OR (Radiograph*[Title/Abstract])OR (Computed tomography[Title/Abstract])OR (CT[Title/Abstract])OR (CAT[Title/Abstract])OR (CTA[Title/Abstract])OR (Computerized axial tomography[Title/Abstract])OR (Magnetic resonance imag*[Title/Abstract])OR (MRI[Title/Abstract])OR (MR[Title/Abstract])OR (Magnetic resonance angio*[Title/Abstract])OR (MRA[Title/Abstract])OR (Scintigraphy[Title/Abstract])OR (DMSA[Title/Abstract])OR (Ultrasound*[Title/Abstract])OR (Sonograph*[Title/Abstract])OR (PET[Title/Abstract])OR (Positron Emission Tomography[Title/Abstract])OR (SPECT[Title/Abstract])OR (Single-photon emission[Title/Abstract])OR (Single photon emission[Title/Abstract])OR (mammogra*[Title/Abstract])	Infan* OR newborn* OR new-born* OR perinat* OR neonat* OR baby OR baby* OR babies OR toddler* OR minors OR minors* OR boy OR boys OR boyfriend OR boyhood OR girl* OR kid OR kids OR child OR child* OR children* OR schoolchild* OR schoolchild OR school child[tiab] OR school child*[tiab] OR adolescen* OR juvenil* OR youth* OR teen* OR under*age* OR pubescen* OR pediatrics[mh] OR pediatric* OR paediatric* OR peadiatric* OR school [tiab] OR school*[tiab] OR prematur* OR preterm*

### Appraise

The two key components of appraisal are validity and strength.^6^ In our example, we chose to only include papers with external validation of results. Once validity has been established, the next step is usually to apply the levels of evidence as defined by the centre for evidence-based medicine.^13^  It is not always necessary to review the primary literature if secondary evidence with a higher level of evidence is available to answer the specific question. However, in newer fields such as radiology AI, there may be less secondary evidence available. Many AI-specific extension documents have been issued such as CLAIM, SPIRIT-AI, and CONSORT-AI^14^ which can aid with the appraisal process. Additionally, we can also systematically appraise the results of the DATO process above (Figure 2).

### Apply

Traditionally, the “apply” phase of the process involves scrutinising the results using, *e.g.* methods such as likelihood ratios, pre-test probability or graphs of conditional probability.^15^ However, further consideration of whether these results are applicable locally is especially important for AI studies. As such, we included only those studies that externally validated their results. Once this has been established, it is important to consider clinical impact and effect on patient outcome.

### Evaluate

**Figure 3.**
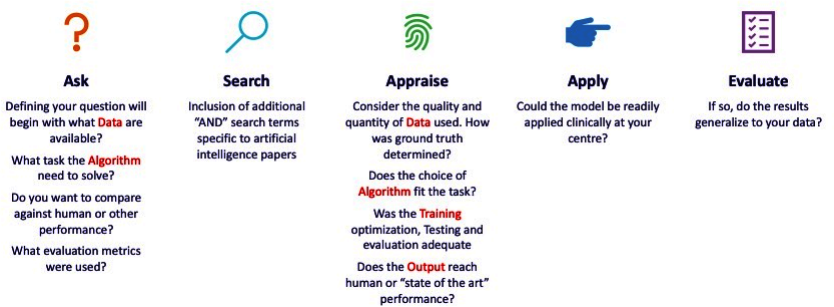
The use of DATO in evidence-based practise. DATO, data algorithm training output.

The purpose of this step is to allow for reflection on the processes and results gleaned from steps 1 to 4 and the potential application to local practise. The use of DATO in the process is illustrated in Figure 3.

## Results

### Search

**Figure 4.**
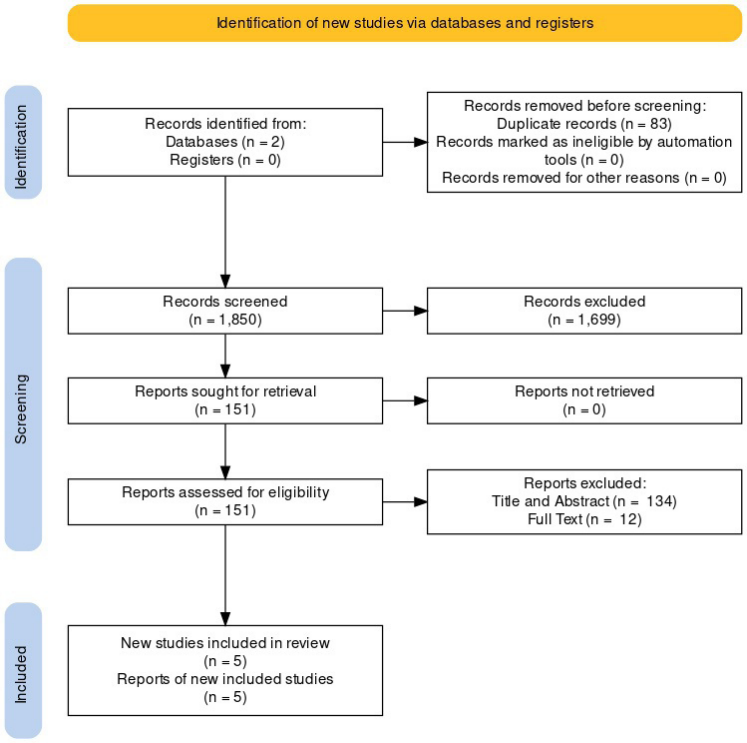
PRISMA flow diagram showing the identification of included papers. PRISMA, Preferred Reporting Items for Systematic reviews and Meta-Analyses.

1933 papers were found (1850 unique), 151 were included as paediatric radiology papers using AI. 17 papers involved bone age estimation of which 5 externally validated their results. Figure 4 shows the relevant The Preferred Reporting Items for Systematic reviews and Meta-Analyses flowchart.

### Appraise

Table 3 lists the five candidate papers that were identified. Following application of the Centre for Evidence Based Medicine (CEBM level) of evidence criteria, four of these were identified as Level 2b papers^13^ (*i.e.* exploratory cohort studies). One paper was a Level 2a study, synthesising 2b studies.^16^ We also used DATO to appraise all five studies. Results for Pan et al^16^are below.

**Table 3.** Candidate papers, selected results and level of evidence

AUTHORS	JOURNAL	STANDARD EROR MONTHS	N	YEAR	STUDY TYPE	CEBM LEVEL OF EVIDENCE
C Spampinato	*MIA*	9.6	1391	2016	Retrospective	2b
Larson	*Radiology*	6	14,036	2017	Retrospective	2b
Mutasa	*JDI*	6.5	10,289	2018	Retrospective	2b
Xuhua Ren	***IEEE***	5.2	12,480	2018	Retrospective	2b
Pan	*Radiology: AI*	3.8	12,611	2019	Systematic Review	2a

### Data

Quality—Pan et al had the highest quality of data as it was highly heterogenous multicentre data.

Quantity—Pan et al also had a comparatively large volume of training data (over 12,000 radiographs).

Ground truth and labelling—Pan et al was based on the weighted average of six experts data and was verified by the RSNA.

### Algorithm

As an ensemble taken from the 24 best-performing models, Pan et al had the only evidence-based model selection.

### Training

For Pan et al Model Development and Model Evaluation, we carried out within the confines of a machine learning challenge and were thus more objective than the other papers. There was a comprehensive validation process using bootstrapping techniques.

### Output

While all appraised papers gave a useful output in terms of age estimation, the Pan paper achieved the best performance.

As the paper with the highest level of evidence and also the paper that had the strongest performing DATO metrics; Pan et al was chosen as the paper of interest.

### Apply

Pan et al^16^ was based on the 2017 RSNA Machine Learning Challenge which was a competition where participants were provided with a set of hand radiographs for determination of bone age, along with bone ages to be used as training data.^17^  The challenge provided a unique opportunity to test the power of ensembling the 48 submitted computer vision models.

Pairs of models were considered to identify the best potential ensembles. The best-performing model was labelled “Model 1” and so on. While most of the models used deep learning, those few that used more traditional machine learning methods (Models 4 and 16 as outlined in Pan et al) were less correlated with the others. It was found that pairs consisting of models with lower individual mean absolute deviations (MAD) did not necessarily outperform other model pairs. Rather, combinations of high performing models with low intermodel correlations (*i.e.* those that err in different ways) tended to achieve better performance.

Individual model performance ranged from MAD of 4.27 to 34.16 months with a median of 5.99 months. The average MAD for the human reviewers was 5.8 months. The ensemble models were able to reduce MAD 3.79 months.  No significant difference was found between the performance of the highest 10 ranking ensembles and human performance.

Therefore, in answer to our question:

“In paediatric patients who require bone age assessment with hand/wrist radiographs, how do AI models compare with human performance using birth certificate as ground truth?”

An ensemble model comprising a deep convolutional neural network combined with an uncorrelated, but high performing simple model (*e.g.* linear model after application Principle Component Analysis), trained on 10,000 well-labelled images should achieve error rates comparable to human performance based on the results of Pan et al.

### Evaluate

This process could be considered successful because it revealed useful potentially information relevant to our question. Combining deep learning methods with simpler, less computationally expensive machine learning techniques may yield better performance than naively combining those with the lowest error. This is because different models err in different ways. Further data would be needed to confirm if this finding generalises beyond the research question asked in this paper.

## Discussion

We have demonstrated who DATO can complement the EBR framework. We asked an answerable question and used DATO to appraise all five studies. Pan et al^16^ had the strongest data with the highest total sample size and most robust data quality measures. They used ensemble models and therefore had the only evidence-based algorithm. The training process for all models was clearly illustrated and they achieved a performance comparable to expert human readers.

The DATO model is intended to be a tool to enable radiologists and related professions systematically assess AI research and products. Due to the rapid expansion of the literature in this area, DATO sits within a busy space, and any methods for reviewing the clinical AI literature exist such as PICO-AI, ML-PICO and the CONSORT, SPIRIT AI extensions, ECLAIR and RSNAs CLAIM checklist.^3,14^ A key advantage of the DATO is how it complements without attempting to replace the PICO or EBP systems. Once a PICO question has been asked, the next question may be “is artificial intelligence appropriate for this question?”. DATO is an appropriate way to answer this question. It places data at the beginning which is how most data science projects start and moves in order through the basic steps of the data science process. It gives clinicians an easy to use starting point to enable meaningful discussion with AI researchers and vendors.

## Conclusion

Using the DATO method to supplement EBR may provide radiologists and related professionals with a systematic approach for reviewing the radiology AI literature enabling for a bottom-up approach to making evidence-based decisions.

## References

[1] Rimmer A . Radiologist shortage leaves patient care at risk, warns Royal College. BMJ 2017; 359: j4683. doi: 10.1136/bmj.j4683 29021184

[2] De Fauw J, Ledsam JR, Romera-Paredes B, Nikolov S, Tomasev N, Blackwell S, et al . Clinically applicable deep learning for diagnosis and referral in retinal disease. Nat Med 2018; 24: 1342–50. doi: 10.1038/s41591-018-0107-6 30104768

[3] Kelly BS, Judge C, Bollard SM, Clifford SM, Healy GM, Aziz A, et al . Radiology artificial intelligence: a systematic review and evaluation of methods (raise). Eur Radiol 2022; 32: 7998–8007. doi: 10.1007/s00330-022-08784-6 35420305 PMC9668941

[4] Keane PA, Topol EJ . With an eye to AI and autonomous diagnosis. NPJ Digit Med 2018; 1(): 40. doi: 10.1038/s41746-018-0048-y 31304321 PMC6550235

[5] Malone DE . Evidence-Based practice in radiology: an introduction to the series. Radiology 2007; 242: 12–14. doi: 10.1148/radiol.2421060010 17185657

[6] Lavelle LP, Dunne RM, Carroll AG, Malone DE . Evidence-Based practice of radiology. Radiographics 2015; 35: 1802–13. doi: 10.1148/rg.2015150027 26466187

[7] Willemink MJ, Koszek WA, Hardell C, Wu J, Fleischmann D, Harvey H, et al . Preparing medical imaging data for machine learning. Radiology 2020; 295: 4–15. doi: 10.1148/radiol.2020192224 32068507 PMC7104701

[8] Greulich WW, Pyle SI . Radiographic atlas of skeletal development of the hand and wrist. The American Journal Of The Medical Sciences 1959; 238: 393. doi: 10.1097/00000441-195909000-00030

[9] Martin DD, Wit JM, Hochberg Z, Sävendahl L, van Rijn RR, Fricke O, et al . The use of bone age in clinical practice-Part 1. Horm Res Paediatr 2011; 76: 1–9. doi: 10.1159/000329372 21691054

[10] Halabi SS . Taking matters into your own hands. Radiol Artif Intell 2020; 2(): e200150. doi: 10.1148/ryai.2020200150 33939791 PMC8082398

[11] Staunton M . Evidence-Based radiology: steps 1 and 2 -- asking answerable questions and searching for evidence. Radiology 2007; 242: 23–31. doi: 10.1148/radiol.2421052135 17185659

[12] Kelly B, Judge C, Bollard SM, Clifford SM, Healy GM, Yeom KW, et al . Radiology artificial intelligence, a systematic evaluation of methods (raise): a systematic review protocol. Insights Imaging 2020; 11(): 133. doi: 10.1186/s13244-020-00929-9 33296033 PMC7726044

[13] Durieux N, Vandenput S, Pasleau F . OCEBM levels of evidence system. [OCEBM levels of evidence system]. Rev Med Liege 2013; 68: 644–49. 24564030

[14] Liu X, Rivera SC, Moher D, Calvert MJ, Denniston AK, Ashrafian H . Reporting guidelines for clinical trial reports for interventions involving artificial intelligence: the CONSORT-AI extension. BMJ 2020; 370: m3164. doi: 10.1136/bmj.m3164 32909959 PMC7490784

[15] Dodd JD . Evidence-Based practice in radiology: steps 3 and 4 -- appraise and apply diagnostic radiology literature. Radiology 2007; 242: 342–54. doi: 10.1148/radiol.2422051679 17255406

[16] Pan I, Thodberg HH, Halabi SS, Kalpathy-Cramer J, Larson DB . Improving automated pediatric bone age estimation using ensembles of models from the 2017 RSNA machine learning challenge. Radiol Artif Intell 2019; 1(): e190053. doi: 10.1148/ryai.2019190053 32090207 PMC6884060

[17] Halabi SS, Prevedello LM, Kalpathy-Cramer J, Mamonov AB, Bilbily A, Cicero M, et al . The RSNA pediatric bone age machine learning challenge. Radiology 2019; 290: 498–503. doi: 10.1148/radiol.2018180736 30480490 PMC6358027

